# High-Precision Positioning Stage Control Based on a Modified Disturbance Observer

**DOI:** 10.3390/s24020591

**Published:** 2024-01-17

**Authors:** Hui Wang, Qiang Li, Feng Zhou, Jingxu Zhang

**Affiliations:** 1 Changchun Institute of Optics, Fine Mechanics and Physics, Chinese Academy of Sciences, Changchun 130033, China; liq@ciomp.ac.cn (Q.L.); zhoufeng@sklao.ac.cn (F.Z.); zhangjx@ciomp.ac.cn (J.Z.); 2 University of Chinese Academy of Sciences, Beijing 100049, China

**Keywords:** decoupling, disturbance suppression, positioning control, voice coil motor

## Abstract

High-precision positioning systems play a crucial role in various industrial applications. This study focuses on improving the performance of a high-precision multi-degrees-of-freedom (DOF) stage. In terms of the controller design, the following two key challenges must be addressed: the cross-decoupling of different DOFs and the impact of external disturbances. To address these problems, a self-tuning approach is proposed for simultaneous decoupling and disturbance suppression. Initially, the stage undergoes static decoupling using a data-based approach, facilitating feedback control for each DOF through single-input, single-output controllers. Addressing dynamic coupling and external disturbance challenges, we introduced a comprehensive evaluation index and a self-tuning multi-input, multi-output disturbance observer. This approach enabled the evaluation and optimization of the disturbance compensation for all DOFs, ensuring optimal positioning accuracy. Finally, we tested the proposed method using a high-precision multi-DOF stage with a real-time control platform. The results demonstrated a significant reduction in the standard deviations of positioning errors in the rx, ry, and z directions by 46%, 58%, and 6%, respectively. The approach used in this study opens avenues for advancements in the design and control of complex multi-DOF systems.

## 1. Introduction

**Figure 1:**
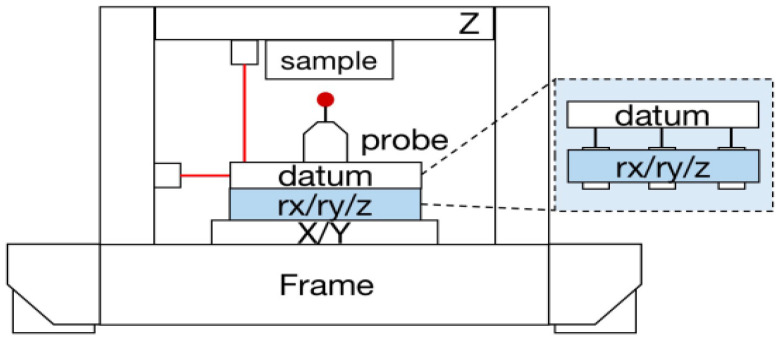
Concept of a high-precision rx/ry/z stage utilized in coordinate measurement devices.

High-precision positioning systems play a crucial role in various industrial applications, including atomic force measurements [1,2], optical alignment systems [3], and semiconductor manufacturing [4,5]. Continued research into precision control techniques is essential to enhance the precision, accuracy, and response speeds of such devices [6,7]. This study focuses on improving the performance of a high-precision rx/ry/z stage used in coordinate measurement devices (see Figure 1), enabling precise *z* axis movement for triggering probes and ensuring stability across the rx-, ry-, and z-axes.

Two critical challenges in controller design must be addressed. First, the inevitable interaction between various degrees of freedom (DOF) arises from the spatial layout of the stage, the different dynamics of the actuators, or assembly errors. For such a multi-input, multi-output (MIMO) system, a static decoupling approach is typically applied in the industry, enabling the use of single-input single-output (SISO) controllers in multiple loops [8,9]. To address the remaining MIMO crosstalk in the entire system, researchers have explored data-based dynamic decoupling approaches and optimization algorithms [10,11].

Moreover, positioning systems are susceptible to external disturbances such as electrical noise and vibrations. In precision positioning stages, flexible mechanisms with low rigidity and natural frequency are typically selected as transmission devices to mitigate Coulomb friction and transmission gaps [12]. However, this design choice results in the limited bandwidth growth of the control system. Therefore, anti-disturbance methods, designed independently of the feedback controller, are applied, including the disturbance observer (DOB) [13,14] and expanded state observer [15,16].

In practice, the parameter tuning of controllers is a complex task. To address these challenges, this study proposes a data model-based self-tuning approach to achieve simultaneous decoupling and anti-disturbance control.

The stage is initially regarded as a linear system operating near the working point in space and adhering to the principle of linear superposition. Subsequently, a proportional matrix is computed to realize motion decoupling, enabling the design of SISO controllers for each DOF. The remaining crosstalk, uncertainty, and external disturbances are amalgamated and treated as centralized disturbances within the system. This allows the DOB to estimate and actively compensate for disturbances in each DOF. In particular, the disturbance compensation for one DOF may affect the other DOFs, introducing new crosstalk. Therefore, the DOB of a MIMO system should be tuned holistically according to the entire system. In the literature, an evaluation index and optimization algorithm based on input and output data are combined to enhance performance control [17]. Witvoet [18] proposed an automated control strategy to overcome the tradeoff between the robustness and optimal performance of large telescopes using an adapted filter. Moreover, Heertjes et al. [10] employed a decoupling structure to optimize the cross-coupling of the active vibration isolation. In this paper, we introduce a novel approach to address complex challenges in high-precision positioning systems. Our method integrates static decoupling, SISO controllers, and a data model-based self-tuning mechanism, offering a holistic solution for simultaneous MIMO crosstalk and disturbance control. The paper introduces an adaptively tuned MIMO disturbance observer (DOB) with an FIR filter matrix and a comprehensive index, ensuring well-balanced disturbance compensation across multi-DOFs. Our iterative approach enhances each DOF’s performance, gradually converging errors in the closed-loop state and significantly improving the overall control performance. To enhance system robustness, we employed a variable-step-size iteration process, adding an adaptive dimension to our methodology. Tailored for high-precision positioning systems, our approach demonstrates adaptability and advanced features, contributing to achieving simultaneous decoupling and anti-disturbance control for complex multi-DOF high-precision positioning systems.

The remainder of this paper is organized as follows. Section 2.1 introduces the plant model and system identification of the multi-DOF stage, and Section 2.2 presents the principle of the proposed self-tuning MIMO DOB. Furthermore, multistage experiments are conducted, and the test results are presented in Section 3, demonstrating the feasibility of this approach. Finally, Section 4 concludes this study.

## 2. Materials and Methods

### 2.1. Plant Description

#### 2.1.1. Lumped-Mass Model and Static Decoupling

**Figure 2:**
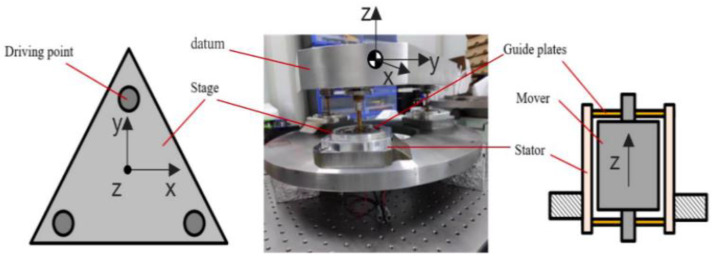
Schematic of the multi-DOF stage driven by VCMs.

The actuator for the positioning stage is a voice coil motor (VCM), selected for its high-precision and fast dynamic characteristics [19]. Moreover, it boasts a larger stroke compared to a piezoelectric ceramic driver. To ensure structural stability, VCMs were strategically arranged in parallel with flexible guide plates positioned between the stators and movers, as illustrated in Figure 2. As the system linearly approximates the working position, motion decoupling can be achieved by controlling the resultant force of the VCMs [20]. Based on this concept, we established a lumped-mass model, capturing the low-frequency dynamics of each DOF, as illustrated in (1) and (2).

(1) $$G_{\left(rx,ry\right)}(s)=\frac{X_{\left(rx,ry\right)}}{T_{\left(rx,ry\right)}}=\frac{1}{J_{\left(rx,ry\right)}s^{2}+C_{\left(rx,ry\right)}s+K_{\left(rx,ry\right)}}$$

(2) $$G_{z}(s)=\frac{X_{z}}{F_{z}}=\frac{1}{M_{z}s^{2}+C_{z}s+K_{z}}$$

Here, $G_{\left(rx,ry\right)}(s)$ and $G_{z}(s)$ represent the transfer function governing the rotation in the x and y directions and translation in the z direction. $X_{\left(rx,ry\right)}$ is the resultant torque in the rotation direction. $T_{\left(rx,ry\right)}$ corresponds to the displacement in each degree of freedom. $J_{\left(rx,ry\right)}$, $C_{\left(rx,ry\right)}$, and $K_{\left(rx,ry\right)}$ denote the equivalent concentrated mass moment of inertia, the damping coefficient, and the stiffness coefficient, respectively. $X_{z}$ is the resultant force in the translation direction. $F_{z}$ corresponds to the displacement in the translational degree of freedom. $M_{z}$, $C_{z}$, and $K_{z}$ denote the equivalent concentrated mass, damping coefficient, and stiffness coefficient in the translational direction, respectively. The system model can be expressed in matrix form, as shown in (3).

(3) $$X^{T}={\left[rx,ry,z\right]}^{T}=\left[\begin{array}{ccc} G_{rx}(s) & 0 & 0\\ 0 & G_{ry}(s) & 0\\ 0 & 0 & G_{z}(s) \end{array}\right]\left[\begin{array}{c} T_{x}\\ T_{y}\\ T_{z} \end{array}\right]=G_{m}(s)F^{T}(s)$$

Herein, $G_{m}(s)$ represents the combined transfer function matrix and $F^{T}(s)$ represents the vector of applied forces. The individual components of the matrix $G_{m}(s)$ are the transfer functions $G_{rx}(s)$, $G_{ry}(s)$, and $G_{z}(s)$ representing the rotational dynamics about the x axis, y axis, and translational dynamics about the z axis, respectively. The system is a MIMO system, and the relationship between the forces applied by each leg and the resulting centralized force can be expressed using (4).

(4) $$F^{T}=K_{0}F_{g}^{T}$$

where $F_{g}^{T}$ represents the output force for each leg and $F^{T}$ is the resultant force. $K_{0}$ is a conversion matrix describing the contribution of the force from each actuator to the resultant force.

**Figure 3:**
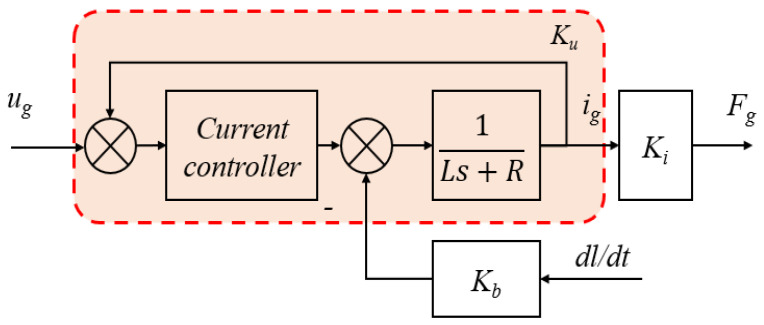
Current loop control [21].

The working principle of the VCMs can be described using (5),

(5) $$\left\{\begin{array}{l} u_{g}^{T}=K_{b}\frac{d\chi_{g}^{T}}{dt}+Ri_{g}^{T}+L\frac{di_{g}^{T}}{dt}\\ F_{g}^{T}=K_{i}\times i_{g}^{T} \end{array}\right.$$

where $K_{b}$ is the back electromotive force (BEF), $K_{i}$ is the thrust coefficient, and $\chi_{g}^{T}$ is the displacement vector of the motor in its direction. *R* and *L* represent the coil resistance and inductance, respectively, in the diagonal matrix form. $i_{g}^{T}$ and $u_{g}^{T}$ represent the input current and voltage vectors, respectively. To ensure the stability of the current and offset the influence of the BEF, a current loop control is introduced, as illustrated in Figure 3, with a high bandwidth, and the model can be simplified as expressed in (6).

(6) $$F_{g}^{T}=K_{i}K_{u}u_{g}^{T}$$

where $K_{u}$ represents the closed-loop static gain of the current loop, as shown in Figure 3.

Accordingly, (3) can be transformed into (7).

(7) $$X^{T}=G_{m}(s)K_{0}K_{i}K_{u}u_{g}^{T}$$

The decoupling matrix *D* and the input *U* satisfy the following expression:

(8) $$u_{g}^{T}=DU^{T}$$

where $U^{T}={\left[U_{z},U_{rx},U_{ry}\right]}^{T}$ denotes the corresponding input for each DOF. The matrix *D* is introduced for decoupling purposes, aiming to mitigate the cross-coupling effects between different DOFs. Its role is to isolate the input contributions for each DOF, providing a clearer representation of the system dynamics. Accordingly, the decoupling model is expressed as (9).

(9) $$G_{d}=\frac{X}{U}=G_{m}(s)K_{0}K_{i}K_{u}D$$

These equations present a general formula. In static scenarios, a data-driven decoupling approach is applied for motion decoupling. Various sets of constant voltages $u_{N}^{T}=[u_{g1}^{0},u_{g2}^{0},...,u_{gn}^{0}{]}^{T}$ are applied to VCMs, and the resulting stable displacements $X_{N}^{T}=[X_{1}^{0},X_{2}^{0},...,X_{n}^{0}{]}^{T}$ are measured. The steady response matrix ${G_{m}(s)K}_{0}K_{i}K_{u}$ is then calculated based on the input and output data. Assuming a diagonal dominance for matrix $G_{d}$, the decoupling matrix *D* can be determined through calculations. This matrix is instrumental in mitigating cross-coupling effects, providing a clearer representation of the system dynamics, and enhancing the control system design.

#### 2.1.2. System Identification and SISO Controller Design

**Figure 4:**
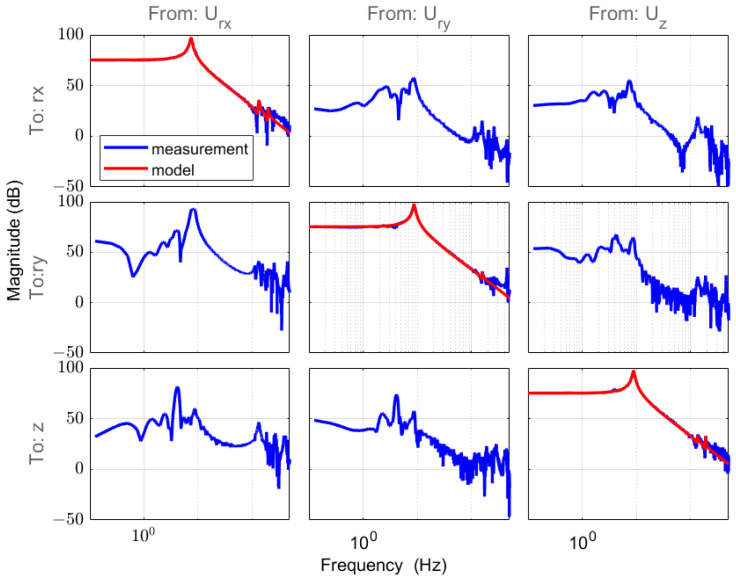
Frequency magnitude response matrix in MIMO form (rx, ry, and z) through the measurement data (blue) and model in the diagonal line (red).

Following static decoupling, we proceeded with system identification and the design of SISO controllers for each DOF. A pseudo-random sequence was employed to excite each DOF, and the transfer functions were obtained using the MATLAB system’s identification toolbox [22]. The model frequency–response characteristics are plotted in Figure 4, providing insights into the dynamic behavior of the system.

For illustration, consider the transfer function in the Z-direction, which is expressed as follows:

(10) $$\frac{X_{z}}{U_{z}}=\frac{1.295\times 10^{7}}{s^{2}+3.701s+2221}$$

Due to the existence of flexible connections, the system is also accompanied by mechanical resonance. The model with mechanical resonance can be approximately expressed as a series connection of a low-order model and a resonance model as in Equation (11) [23]:

(11) $$f_{i}=\frac{s^{2}+\zeta_{ni}\omega_{ni}s+\omega_{ni}^{2}}{s^{2}+\zeta_{di}\omega_{di}s+\omega_{di}^{2}}\frac{\omega_{di}^{2}}{\omega_{ni}^{2}}$$

where $\zeta_{ni}$ and $\zeta_{di}$ represent the damping ratio of the low-order model and the resonance model. $\omega_{ni}$ and $\omega_{di}$ represent the natural frequency of the low-order model and the resonance model. 

Considering mechanical resonance, the transfer function is expressed as follows:

(12) $$\frac{X_{z}}{U_{z}}=\frac{2.769\times 10^{8}s^{2}+1.45\times 10^{10}s+1.772\times 10^{14}}{19.41s^{4}+765.6s^{3}+1.373\times 10^{7}s^{2}+5.218\times 10^{7}s+3.038\times 10^{10}}$$

Following this, we implemented the proportional–integral–derivative (PID) control strategy for each DOF. The PID controller, a staple in control system engineering, is formulated as follows:

(13) $$C=K_{p}+\frac{K_{i}}{s}+\frac{K_{d}s}{T_{f}s+1}$$

where *C* represents the output and $K_{p}$, $K_{i}$, $K_{d}$ are the proportional, integral, and derivative gains, with $T_{f}$ acting as the filter time constant for the derivative term.

**Figure 5:**
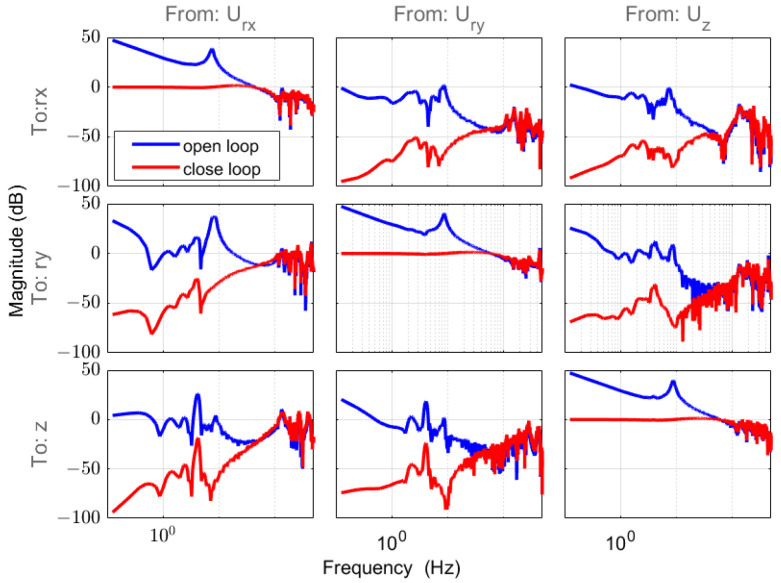
Frequency magnitude response matrix in MIMO form (rx, ry, and z) with designed SISO controllers for every DOF in an open loop (blue) and a closed loop (red).

The frequency response, illustrated in Figure 5, demonstrates the realization of motion control for the multi-DOF stage through the application of static decoupling via the SISO controller.

### 2.2. Dynamic Decoupling through MIMO DOB

#### 2.2.1. DOB Design

Although SISO controllers offer a solution for each DOF using static decoupling, the remaining dynamic crosstalk and external disturbances (electric noise and vibration) can reduce the positioning stage accuracy. By considering dynamic coupling and external disturbances as lumped disturbances, we employed the DOB to achieve simultaneous dynamic decoupling and disturbance suppression.

**Figure 6:**
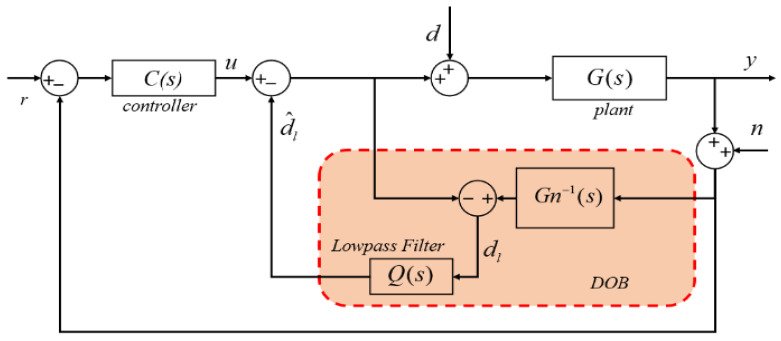
Schematic of DOB [28].

The DOB, pioneered by Ohnishi et al. [24,25,26,27], serves to estimate and actively compensate for equivalent disturbances when direct testing of the disturbance is impractical. The primary principle is illustrated in Figure 6, where *G* and *Gn* represent the plant and nominal models of the system, respectively; *d* and *n* are the equivalent external disturbance and measurement noise, respectively.

From the block diagram, the equivalent concentrated disturbance is given by (14)

(14) $$d_{l}(s)=(G{\left(s\right)}^{-1}-Gn{\left(s\right)}^{-1})y(s)+d(s)+Gn{\left(s\right)}^{-1}n(s)$$

This comprises the difference between the plant and nominal models, external disturbances, and measurement noise [13]. According to Figure 6, after active compensation via the filter *Q*, the system output is expressed as follows:

(15) $$y=G_{uy}(s)u+G_{dy}(s)d-G_{ny}(s)n\;\left\{\begin{array}{l} G_{uy}=\frac{\frac{1}{1-Q(s)}G(s)}{1+\frac{1}{1-Q(s)}G(s)Gn^{-1}(s)Q(s)}=\frac{G(s)Gn(s)}{Gn(s)+Q(s)(G(s)-Gn(s))}\\ G_{dy}=\frac{G(s)}{1+\frac{1}{1-Q(s)}G(s)Gn^{-1}(s)Q(s)}=\frac{G(s)Gn(s)(1-Q(s))}{Gn(s)+Q(s)(G(s)-Gn(s))}\\ G_{ny}=\frac{\frac{1}{1-Q(s)}G(s)Gn^{-1}(s)Q(s)}{1+\frac{1}{1-Q(s)}G(s)Gn^{-1}(s)Q(s)}=\frac{G(s)Q(s)}{Gn(s)+Q(s)(G(s)-Gn(s))} \end{array}\right.$$

where $G_{uy}$, $G_{dy}$, and $G_{ny}$ represent the transfer function from the output of the controller to the system output, from the disturbance input to the system output, and from the noise input to the system output, respectively. From (15), when *Q* equals one, $y\;\approx \;G_{n}(s)u+n$, which implies that the system outputs are close to the outputs of the nominal model. Therefore, the bandwidth of *Q* determines the bandwidth of the DOB. If Δ(s) is considered to represent the product perturbation of the nominal object, the relationship between *G* and *Gn* can be written as (16).

(16) $$G(s)=Gn(s)\left(1+\Delta (s)\right)$$

To meet the robustness requirements of the inner loop, it must be guaranteed according to the principle of small gain in (17), as follows:

(17) $${\left\|Q(j\omega )\Delta (j\omega )\right\|}_{\infty }\leq 1$$

The system’s uncertainty rises with frequency, indicating that the bandwidth of the filter cannot increase indefinitely. Generally, a filter is designed as follows: [29]

(18) $$Q(s)=\frac{\sum_{k=0}^{M}\alpha_{k}{\left(\tau s\right)}^{k}}{M{\left(\tau s+1\right)}^{N}},\;\alpha_{k}=\frac{N!}{\left(N-k\right)!k!}$$

where τ is the time constant of the filter, and *N* and *M* are the orders of the numerator and denominator, respectively. The value range of *k* is (1, 2…*n*), such that the relative order of *Q* is greater than the relative order of *Gn*, ensuring that the inverse of *Gn* can be physically implemented. Based on this analysis, the filter design determines the interference suppression effect and affects the robustness and stability of the system.

#### 2.2.2. Self-Tuning MIMO DOB

According to the DOB principle, to ensure robustness, the higher the bandwidth of the low-pass filter, the stronger the disturbance suppression ability [30]. Because of the existence of dynamic coupling, when disturbance compensation is performed on one DOF, unnecessary inputs can be generated for other DOFs. This implies that the DOB in one DOF improves the performance of that DOF but may reduce the performance of others. Moreover, the mutual coupling between the different DOFs varies with the magnitude of the external disturbance. Therefore, the bandwidth of the DOBs in different DOFs must be well balanced to enable the overall MIMO system to achieve an optimal anti-disturbance ability with the best performance.

**Figure 7:**
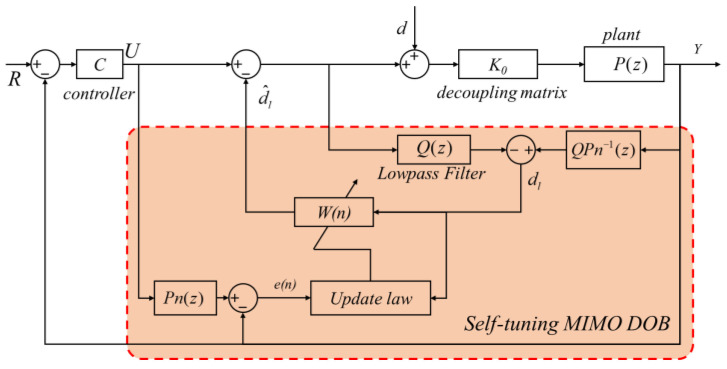
Self-tuning MIMO DOB.

To coordinate the disturbance compensation of various DOFs and realize integrated active interference compensation, a MIMO DOB structure is proposed, as visualized in Figure 7. By setting a comprehensive evaluation index to describe the performance, the parameters can be self-tuned based on the input and output data.

In Figure 7, *Q* is the filter matrix form, as in (19), in which each element is designed in the same form as (18).

(19) $$Q=\mathrm{diag}\left(Q_{1}(z),Q_{2}(z),Q_{3}(z)\right)$$

In most applications, filters are designed with a high bandwidth, satisfying the requirements of relative order. The performance and robustness of the MIMO DOB are determined using the FIR filter matrix *W*, which is optimized after continuous iterations. The structure of *W* is as follows:

(20) $$W=\left[\begin{array}{ccc} W_{11} & 0 & 0\\ 0 & W_{22} & 0\\ 0 & 0 & W_{33} \end{array}\right]$$

(21) $$W_{ij}=\theta_{0}+\theta_{1}^{ij}z^{-1}+\theta_{2}^{ij}z^{-2}+\theta_{N}^{ij}z^{-N}$$

Herein, *N* represents the order of the Wiener filter and θ is the filter coefficient. Choosing a large *N* can increase the changing speed of the gain in the frequency domain, but it also increases the computational complexity and increases the lag. In this article, *N* is set to be the same as the order of *Q*. After the filter matrix, the estimated value of the equivalent disturbance is given as (22).

(22) $$d_{l}^{T}(n)=\sum_{k=0}^{N}W(n)d_{l}^{T}(n-k)$$

For the adaptive filter, the error signal represents the difference between the reference and actual signals, as shown in (23). As the DOB brings the system closer to the nominal model after active compensation, the error of the disturbance estimation can be expressed as the difference between the actual output and the nominal model output, as in (24). When *e*(*n*) converges, the equivalent disturbance is suppressed, and the system performance improves.

(23) $$e^{T}(n)={\hat{d}}_{l}^{T}(n)-d_{l}^{T}(n)=W(n)d_{l}^{T}(n)-d_{l}^{T}(n)$$

(24) $$e^{T}(n)=Y(n)-PnU^{T}(n)$$

The comprehensive evaluation function is selected as follows:

(25) $$J(n)=e(n)\gamma e{\left(n\right)}^{T}\;\mathrm{where}\;\left\{\begin{array}{l} e(n)=\left[e_{z}^{T}(n),e_{rx}^{T}(n),e_{ry}^{T}(n)\right]\\ \gamma =\mathrm{diag}\left[\gamma_{z},\gamma_{rx},\gamma_{ry}\right] \end{array}\right.$$

Herein, γ is the diagonal weighting matrix. The weight coefficient of the filter adopts the steepest descent method iteration, as expressed in (26).

(26) $$W(n+1)=W(n)+\mu \left(-\frac{\partial J(n)}{\partial W(n)}\right)$$

When using the least mean square algorithm, we obtained the gradient ∂J(n)/∂W(n) based on test data, as expressed in (27) and (28), where *μ* is the step length of the iteration.

(27) $$-\frac{\partial J(n)}{\partial W(n)}=2\gamma e{\left(n\right)}^{T}d_{l}(n)$$

(28) $$W(n+1)=W(n)+2\mu e(n)\gamma d_{l}^{T}(n)$$

**Figure 8:**
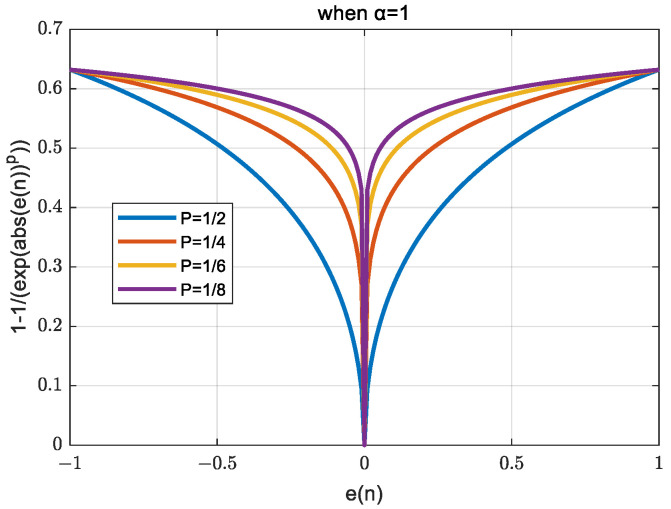
Waveforms of improved step function [31].

To ensure the robustness of the system, a soft threshold method can be used to constrain the filter [18]. Moreover, in this study, a variable step-length approach is introduced. In an iterative process, a large step length can lead to poor system robustness or even system instability, whereas a small step length can decelerate the convergence. Therefore, a larger step length that can stabilize the system is selected as the initial state. With the convergence of the error, the stability margin of the system decreases gradually, and the step length is reduced to ensure the robustness of the system. The variable step-size function and its waveform are shown in (29) and Figure 8, respectively

(29) $$\mu (n)=\alpha \left(1-\frac{1}{\exp\left({\left|e(n)\right|}^{p}\right)}\right),p=\frac{1}{2},\frac{1}{4},\frac{1}{6},\cdots$$

Although *e*(*n*) cannot converge to zero in practice, a certain allowable margin $e_{m}$ for *e*(*n*) is set as follows:

(30) $$e(n)=\left\{\begin{array}{ll} e(n), & \mathrm{if}\;\left|e(n)\right|>e_{m}\\ 0, & \mathrm{otherwise} \end{array}\right.$$

Finally, after tuning α and σ, the weight coefficients are subject to iteration, as expressed in (31).

(31) $$\begin{array}{c} W(n+1)=W(n)+2\alpha (1-\frac{1}{\exp({\left|e_{m}(n)\right|}^{p})})e_{m}(n)\gamma d_{l}^{T}(n),\\ s.t.\left\{\begin{array}{ll} p=\frac{1}{2},\frac{1}{4},\frac{1}{6},\ldots , & \\ e_{m}(n)=e(n), & \left|e(n)\right|>\sigma \\ e_{m}(n)=0, & \left|e(n)\right|\leq \sigma \end{array}\right. \end{array}$$

## 3. Results

The tests were performed on an NI-PXI platform at a sample rate of 3 kHz. The positioning stage shown in Figure 2 is placed on the vibration isolation table. The vibration isolation grade of the vibration isolation table in the horizontal direction is about VC-E, and the vibration isolation grade in the vertical direction is approximately VC-F. At the same time, the control loop of the positioning stage is also affected by the electrical noise of the driver and the measurement noise of the sensor. All disturbances are real-time and dynamic, resulting in the positioning error of the positioning stage.

The SISO controllers were designed as described in Section 2. Using the derived transfer functions as the nominal model to design the MIMO DOB, as detailed in Section 2.2.2, the initial value of the filter matrix coefficient was set to zero. Both *α* and *σ* were initially tuned, and self-tuning commenced in the closed-loop state.

**Figure 9:**
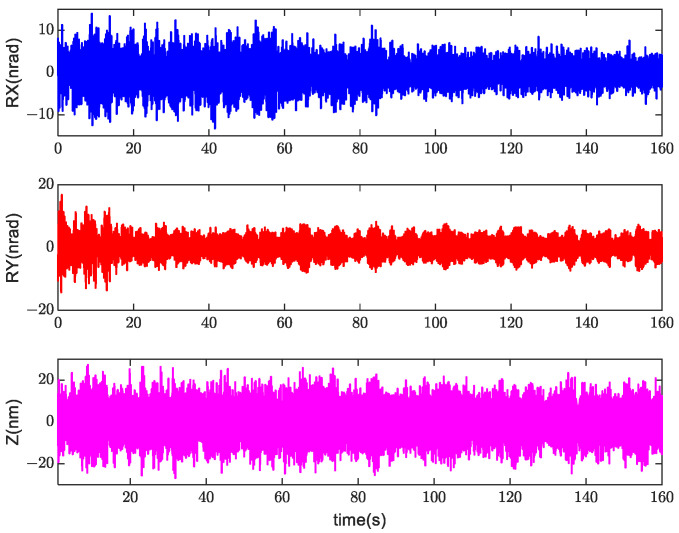
Time-series position error signals during self-tuning.

After adaptive tuning, the filter coefficients were consolidated as part of the controller. The filter coefficients underwent iteration within the control cycle, and the time-domain position error data for the DOFs are plotted in Figure 9. The error converged, indicating that the position accuracy gradually improved. After approximately 140 s, the position error tended to be stable.

**Figure 10:**
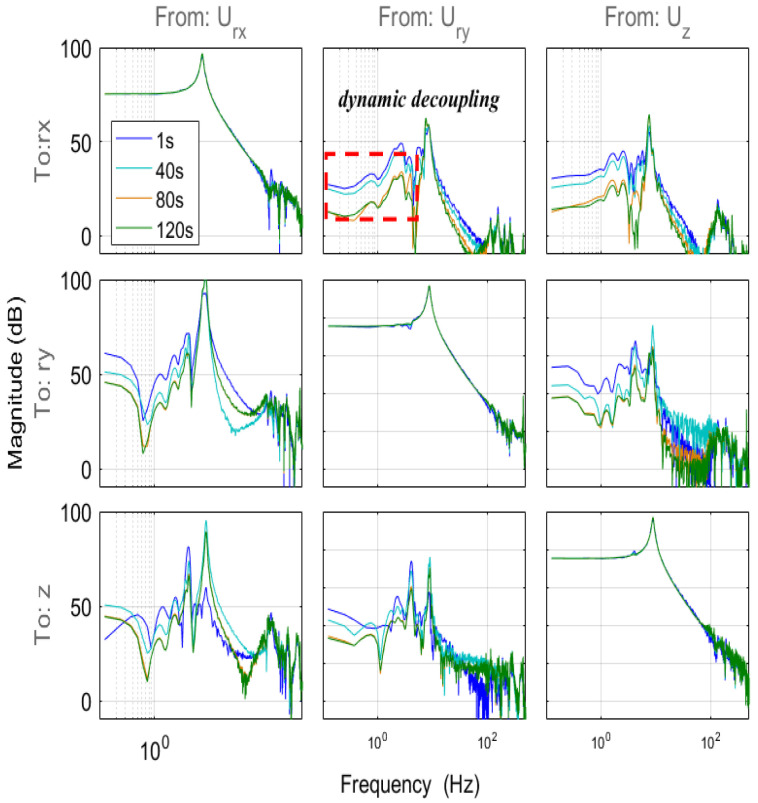
Magnitude response of the system at different iteration times.

The filter coefficients were extracted at 1, 40, 80, and 120 s after the start of the iteration. Through a combination of the filter matrix and magnitude response of the MIMO system, it is possible to compare the models after the active compensation of the adaptive DOB at different iteration periods, as depicted in Figure 10. The proposed approach minimizes dynamic decoupling, with the models on the diagonal line remaining almost constant.

**Figure 11:**
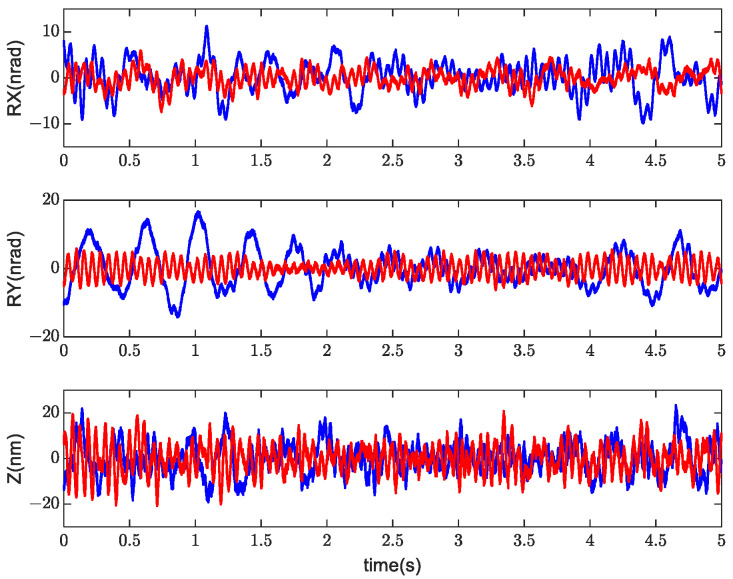
Time-series position error signals (measurement) before (blue) and after (red) self-tuning.

To quantify the improvement in the performance, a comparison of the position error data in the time domain before and after self-tuning is presented in Figure 11.

(32) $$e_{SD}=\sqrt{\frac{\sum_{i=1}^{n}{\left(e_{i}-\left(e_{M}\right)\right)}^{2}}{n}},\;\;(e_{M}=\frac{\sum_{i=1}^{n}e_{i}}{n})$$

The standard deviation in (32) was used to evaluate the position’s precision. Compared with the data before adaptive tuning, as listed in Table 1, the position errors in the rx, ry, and z directions were reduced by 46%, 58%, and 6%, respectively. The improvement effects of different DOFs were different because the positioning error of the stage was not only related to MIMO DOB but also related to the parameter settings of feedback controller C, as shown in Figure 7. It can be proved from experimental results that the proposed approach effectively improves the positioning accuracy of the stage through self-tuning.

## 4. Discussion

This study investigated a self-tuning approach for simultaneous disturbance suppression and decoupling in the position control of a high-precision multi-DOF stage. By utilizing a static decoupling matrix, SISO controllers were designed for MIMO systems, and a data-based decoupling approach was proven effective through open- and closed-loop tests and analysis. The DOB was used to estimate and actively compensate for the combined disturbance, discerning the influence of the residual dynamic crosstalk and external disturbances. The proposed self-tuning MIMO DOB was devised to achieve a balanced multi-DOF disturbance compensation. This method was validated through testing on a high-precision multi-DOF stage. The experimental results indicated successful dynamic decoupling, affirming that the proposed approach effectively improved the positioning accuracy, with the standard deviations of positioning errors in the rx, ry, and z directions reduced by 46%, 58%, and 6%, respectively. This study’s findings suggest that the proposed self-tuning disturbance observer-based control approach significantly enhances the performance of high-precision positioning stages, leading to improved accuracy. The successful application of this approach opens avenues for advancements in the design and control of complex multi-DOF systems, offering potential benefits in various precision engineering applications.

## Figures and Tables

**Table 1:** Quantification of improved performance ($e_{SD}$).

	RX (nrad)	RY (nrad)	Z (nm)
Before adaptive tuning	3.61	5.65	6.88
After adaptive tuning	1.95	2.40	6.44
Reduced position error	46%	58%	6%

## Data Availability

The original contributions presented in the study are included in the article material, further inquiries can be directed to the corresponding author.
